# Design of the Resistance and Endurance exercise After ChemoTherapy (REACT) study: A randomized controlled trial to evaluate the effectiveness and cost-effectiveness of exercise interventions after chemotherapy on physical fitness and fatigue

**DOI:** 10.1186/1471-2407-10-658

**Published:** 2010-11-30

**Authors:** Caroline S Kampshoff, Laurien M Buffart, Goof Schep, Willem van Mechelen, Johannes Brug, Mai JM Chinapaw

**Affiliations:** 1 EMGO Institute for Health and Care Research, department of Public and Occupational Health, VU University Medical Center, Amsterdam, The Netherlands; 2 EMGO Institute for Health and Care Research, department of Epidemiology and Biostatistics, VU University Medical Center, Amsterdam, The Netherlands; 3 Máxima Medical Center, department of Sports Medicine, Veldhoven, The Netherlands

## Abstract

**Background:**

Preliminary studies suggest that physical exercise interventions can improve physical fitness, fatigue and quality of life in cancer patients after completion of chemotherapy. Additional research is needed to rigorously test the effects of exercise programmes among cancer patients and to determine optimal training intensity accordingly. The present paper presents the design of a randomized controlled trial evaluating the effectiveness and cost-effectiveness of a high intensity exercise programme compared to a low-to-moderate intensity exercise programme and a waiting list control group on physical fitness and fatigue as primary outcomes.

**Methods:**

After baseline measurements, cancer patients who completed chemotherapy are randomly assigned to either a 12-week high intensity exercise programme or a low-to-moderate intensity exercise programme. Next, patients from both groups are randomly assigned to immediate training or a waiting list (i.e. waiting list control group). After 12 weeks, patients of the waiting list control group start with the exercise programme they have been allocated to.

Both interventions consist of equal bouts of resistance and endurance interval exercises with the same frequency and duration, but differ in training intensity. Additionally, patients of both exercise programmes are counselled to improve compliance and achieve and maintain an active lifestyle, tailored to their individual preferences and capabilities.

Measurements will be performed at baseline (t = 0), 12 weeks after randomization (t = 1), and 64 weeks after randomization (t = 2). The primary outcome measures are cardiorespiratory fitness and muscle strength assessed by means of objective performance indicators, and self-reported fatigue. Secondary outcome measures include health-related quality of life, self-reported physical activity, daily functioning, body composition, mood and sleep disturbances, and return to work. In addition, compliance and satisfaction with the interventions will be evaluated. Potential moderation by pre- and post-illness lifestyle, health and exercise-related attitudes, beliefs and motivation will also be assessed. Finally, the cost-effectiveness of both exercise interventions will be evaluated.

**Discussion:**

This randomized controlled trial will be a rigorous test of effects of exercise programmes for cancer patients after chemotherapy, aiming to contribute to evidence-based practice in cancer rehabilitation programmes.

**Trial registration:**

This study is registered at the Netherlands Trial Register (NTR2153)

## Background

Cancer treatment has made substantial progress in the last decades. Survival rates after cancer treatment have improved up to 56% in male and 62% in female patients [1]. This is a major achievement; it is, however, important to acknowledge that cancer and cancer treatment are associated with long-term physical and psychosocial side effects. These sequelae include decreased muscle strength, reduced cardiorespiratory fitness, reduced lean body mass, bone loss, severe feelings of fatigue [2,3], depression, emotional distress, anxiety and decreased self-esteem [4]. Fatigue is one of the most common side effects of cancer treatment, affecting approximately 70% of the cancer population receiving radiation therapy and chemotherapy [5,6]. Even years after treatment, feelings of fatigue persist in 30% of cancer patients [7]. This has great impact on the patient's quality of life [5,8].

Cancer rehabilitation programmes have become a great matter of interest as component of cancer patient care to reduce the side effects of cancer treatment and to enhance a patient's quality of life. In the Netherlands, two cancer rehabilitation programmes have been evaluated in previous years. The first programme was based on a biopsychosocial approach, combining physical exercise with psychosocial activities in a group format. Exercise started with low-to-moderate intensities, and workload increased gradually after four weeks training [9]. The second cancer rehabilitation programme focused on high intensity resistance and endurance exercise [10]. Both rehabilitation programmes were well tolerated by most patients and improvements in physical fitness and health-related quality of life were reported, directly after completion of the programmes [10,11] and after respectively 9 [12] and 12 months [13] follow-up.

Systematic reviews of the literature [14-17] underline the positive physical and psychosocial benefits from exercise programmes accordingly. Evidence suggests that exercise may result in improved physical fitness, reduced levels of fatigue and enhanced health-related quality of life. However, the results must be interpreted with caution [14,18]. Overall, the methodological quality of many of the studies reviewed was moderate and opportunities to improve the scientific methodology were evident. Authors of the reviews suggested including larger sample sizes, using appropriate control groups, and using a comparable set of valid and reliable outcome measures in future randomized controlled trials (RCTs). Moreover, certain aspects of the examined exercise programmes were less than optimal: most exercise programmes were relatively short in duration (less than 12 weeks), the programmes did not stimulate the patients to stay physically active after the programme, and studies frequently included aerobic exercises such as walking and cycling, but no resistance exercises.

Accumulating evidence suggests that resistance exercises may have great potential as well [15,19]. A recent systematic review of twenty-four studies evaluating resistance exercise in cancer patients post-treatment [20] reported beneficial effects on cardiorespiratory fitness and muscle strength. Furthermore, the studies included in the review did not report adverse effects, indicating that resistance exercise was well-tolerated. Courneya et al. [21] compared aerobic exercise with resistance exercise in breast cancer patients. While aerobic exercises showed significant improvements in self-esteem, preserved aerobic fitness, and maintained body fat levels, resistance exercises significantly improved self-esteem, muscle strength, and lean body mass. A recent roundtable, organised by the American College of Sports and Medicine (ACSM), came to consensus that both aerobic and resistance exercise are recommended to be prescribed in cancer patients [22].

Current ACSM exercise recommendations for cancer patients include moderate intensity exercises with aerobic exercises at 40% to 60% of heart rate reserve (HRR) three to five times per week for 20 to 60 minutes and resistance exercises at 40% to 60% of one-repetition maximum (1-RM) two or three times per week with one to three sets of 8 to 12 repetitions per exercise [23]. However, the ACSM acknowledges the remaining gap in existing knowledge on the optimal mode, frequency, duration and intensity of exercise [22]. High intensity exercise has shown to improve physical fitness and enhance health-related quality of life in cancer patients who competed chemotherapy [10]. Also in patients with heart failure, high intensity exercise was feasible and resulted in greater improvements in physical fitness as compared to lower intensity exercise [24].

High quality scientific research is needed to firmly establish the range and magnitude of positive effects of exercise programmes among cancer patients and to determine optimal exercise intensities in this population.

This paper presents the design of a randomized controlled multi-centre trial to evaluate the effectiveness and cost-effectiveness of a high intensity exercise programme compared to a low-to-moderate intensity exercise programme and a waiting-list control group on physical fitness (cardiorespiratory fitness and muscle strength), and fatigue in cancer patients who completed chemotherapy. We hypothesize that patients in both exercise programmes will achieve more muscle strength, greater gains in cardiorespiratory fitness and will report lower levels of fatigue compared to the patients who are allocated to the waiting list control group. Furthermore, we hypothesize these improvements to be greater in patients who completed the high intensity exercise programme compared to patients who completed the low-to-moderate intensity exercise programme, both on the short and longer (at one year follow-up) term. Additionally, we compare the cost-effectiveness of the high intensity exercise programme with the low-to-moderate intensity exercise programme.

## Methods

**Figure 1:**
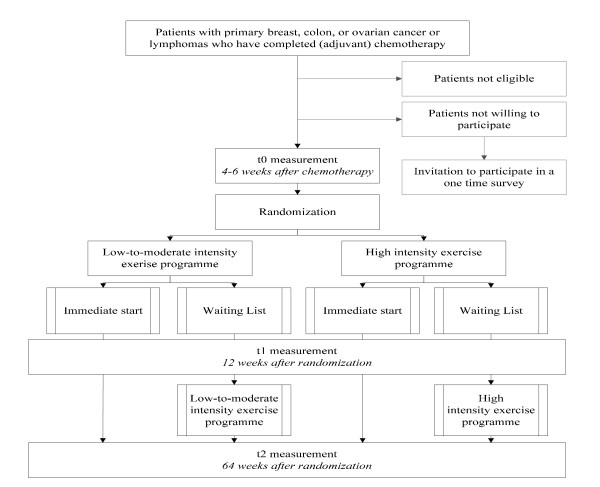
**Design and Procedures of the study**.

The Resistance and Endurance exercise After ChemoTherapy (REACT) study is one of four RCTs included in the Alpe d'HuZes Cancer Rehabilitation (A-CaRe) program [25]. All four studies in this programme have been designed to evaluate the effectiveness and cost-effectiveness of exercise-based rehabilitation programmes in different cancer patient groups. Figure 1 shows the design of the REACT study and the flow of eligible patients through the trial. The Medical Ethics Committee of the Máxima Medical Center approved the study.

### Study sample

Patients with histological confirmed primary breast, colon or ovarian cancer, or lymphomas with no indication of recurrent or progressive disease, who completed (adjuvant) chemotherapy with curative intention, and aged between 18 and 70 years are eligible for this study. Patients who are not able to perform basic activities of daily living such as walking or biking, who show cognitive disorders or severe emotional instability, who are suffering from other disabling comorbidity that might hamper physical exercise (e.g. heart failure, chronic obstructive pulmonary disease (COPD), orthopaedic conditions and neurological disorders), and patients who are unable to understand and read the Dutch language are excluded from the study.

### Recruitment and randomization

The patients are recruited from three hospitals in the southern part of the Netherlands: Máxima Medical Center (Veldhoven/Eindhoven), Catharina Hospital (Eindhoven), and Elkerliek Hospital (Helmond). Expectations are that more hospitals in the southern part of the Netherlands will be invited to collaborate.

In consultation with the treating medical oncologist, the oncology nurse determines if patients in their clinical setting are eligible for the study. All potentially eligible patients receive written information to take home. Next, patients are contacted by telephone and invited to query any question about the study. Patients who are willing to participate are asked to provide written informed consent.

After completing all baseline measurements, patients are stratified by tumour type and hospital and randomly assigned to one of the following groups: 1) high intensity exercise programme or 2) low-to-moderate intensity exercise programme. Next, patients from both groups are randomly assigned to immediate training or waiting list (i.e. waiting list control group). After 12 weeks, patients of the waiting list control group start with the exercise programme they have been allocated to. The research assistant uses statistical software for randomization of the sample and informs patients about the results. Allocation sequence is concealed from the medical team. Study outcomes are assessed by a blinded professional and patients are instructed not to reveal their treatment allocation.

Patients who choose not to participate in the REACT study are asked to complete a one time survey. This questionnaire includes relevant characteristics, the reason for not participating and questions on the current attitudes towards and beliefs about exercise.

### Intervention

This study includes three arms: a high intensity exercise programme, a low-to-moderate intensity exercise programme, and a waiting list control group. Both interventions consist of equal bouts of resistance and endurance interval exercises with similar frequency and duration; the exercise programmes differ in training intensity only.

All patients train in groups of a maximum of eight persons, on specific resistance training equipment and ergometers (e.g. bicycle, treadmill), twice a week for 12 weeks under supervision of a physiotherapist. Additionally, a physical active lifestyle is stimulated in both interventions groups equally, using behavioural motivational techniques (see below; Behavioural motivational counselling programme). The safety of both exercise programmes is guaranteed by a comprehensive intake procedure performed by a sports physician or rehabilitation specialist. Medical history together with possible physical limitations is reported, and if necessary, adaptations in training methods or specific advice to patient and physiotherapists are provided.

#### Intervention A; High intensity exercise programme

The high intensity resistance exercise session consists of six exercises targeting the large muscle groups as follows: 1) vertical row (focusing on m. longissimus, m. biceps brachii, m. rhomboideus); 2) leg press (m. quadriceps, m. glutei, m. gastrocnemius); 3) bench press (m. pectoralis major, m. triceps); 4) pull over (m. pectoralis, m. triceps brachii, m. deltoideus, m. trapezius); 5) abdominal crunch (m. rectus abdominis); 6) lunge (m. quadriceps, m. glutei, hamstring muscles). Resistance exercises are performed at 70-85% of 1-RM and consist of two sets of 10 repetitions. Every four weeks (week 5 and 9) the training progress is evaluated by means of an indirect 1-RM test, and the resistance is adjusted accordingly. The 1-RM is the greatest resistance that can be moved through the full range of motion in a controlled manner with good posture, and is considered to be the standard for dynamic strength assessment [26]. To minimize the risk of injury we apply an indirect 1-RM measurement. Following a warm-up, the physiotherapist estimates a workload at which the patient is expected to perform approximately four to eight repetitions, taking into consideration sex, height and age. In case that the physiotherapist's judgement regarding this workload proves to be incorrect, another assessment will be carried out a little later.

The first four weeks, the high intensity endurance interval exercises consist of two times eight minutes cycling, with alternating workloads: 30 seconds at a workload of 65% of the maximal workload assessed by the steep ramp test and 60 seconds at 30%. From the fifth week onwards, the duration of the latter block is reduced to 30 seconds. Every four weeks, the training progress is evaluated by means of the steep ramp test, and the workload is adjusted accordingly. The steep ramp test is an incremental bicycle ergometer test, in which the patient is instructed to cycle at a rate between 70 and 80 revolutions per minute (RPM), starting at 25 watt (W), after which the load is increased by 25W every 10 seconds. The test ends if cycling rate falls below 60 RPM. The obtained maximal workload during the steep ramp test, indicated as maximal short exercise capacity (MSEC) [27], the time cycled at that load and heart rate (HR) at the end of the test are recorded. The steep ramp test has shown to be a reliable (Intraclass Correlation Coefficient (ICC) = 0.996) and valid (correlation with peakVO_2 _= 0.85) test to estimate maximal workload in cancer patients [27].

From the fifth week onwards, an additional endurance interval session is included in the programme, in exchange for one block of eight minutes cycling. This interval session consists of three bouts of five minutes with one minute of rest in between each bout. During the five minutes of exercise, patients train on ergometers (e.g. cycle ergometer or treadmill) at a constant workload in which the training HR is 80% of their HRR or higher. Training HR is determined by using the Karvonen formula [28], using the maximum heart rate (peak HR) obtained from baseline measurements and heart rate at rest (HR rest).

#### Intervention B; Low-to-moderate intensity exercise programme

Resistance exercise session of the low-to-moderate intensity exercise programme consists of the same six exercises as the high intensity exercise programme, but with lower intensity. All exercises are performed at 40-55% of 1-RM, with a frequency of two sets of 10 repetitions.

The low-to-moderate intensity endurance interval exercises start with two times cycling of eight minutes as well. The alternating workloads are adjusted in 30 seconds at a workload of 45% of the MSEC assessed by the steep ramp test and 60 seconds at 30%. From week five onwards, the duration of the latter block is reduced to 30 seconds in a similar way.

Every four weeks (week 5 and 9) training progress of the resistance and endurance interval exercises are evaluated and adjusted accordingly.

Comparable to the high intensity programme, from the fifth week onwards one block of eight minutes cycling is exchanged by an additional endurance interval session which consists of three bouts of five minutes, with one minute of rest in between each bout. Patients who follow the low-to-moderate exercise programme should achieve 40-50% of their HRR during these three bouts of five minutes of exercise at a constant workload.

#### Behavioural motivational counselling programme

A behavioural motivation component is included to improve compliance and stimulate physical activity outside the exercise programme. Patients are encouraged to be moderately physically active for at least 30 minutes, three times per week in addition to the supervised exercise programme. After completion of the 12-week exercise intervention, patients are encouraged to maintain an active lifestyle with the aim to be moderately physically active for at least 30 minutes three times per week as well as to continue with physical exercises at a higher intensity level for at least 20 minutes two times per week [23]. Specific programme elements include the provision of general and motivational information about physical activity, both verbally and via folders, and discussing individual barriers and facilitators. Behavioural motivational counselling is offered by the physiotherapist in close collaboration with the sports physician or rehabilitation specialist.

#### Waiting list control group

The control arm of this trial consists of a waiting list in order to control for spontaneous recovery over time. Patients from the waiting list control group start either with the high intensity exercise programme or the low-to-moderate intensity exercise programme after 12 weeks.

### Study Outcomes

All studies within A-CaRe Clinical Research use similar methodologies and a comparable set of outcome measures [25]. Within the REACT study, primary and secondary outcome measures are assessed at baseline (t = 0) at the time of inclusion in the trial (4-6 weeks after ending chemotherapy), 12 weeks after randomization (t = 1), and 64 weeks after randomization (t = 2). For logistic reasons, all physical tests are conduced centrally at Máxima Medical Center in Eindhoven. All professionals follow detailed and standardized test protocols. The questionnaires can be completed at home or via internet.

### Primary outcome measures

#### Cardiorespiratory fitness

Cardiorespiratory fitness is measured during a maximal exercise test on an electronically braked cycle ergometer according to a ramp protocol [29], in which the resistance gradually increases every six seconds aiming to achieve the maximum within 8 to 12 minutes. All patients are instructed to cycle with a pedal frequency between 70 and 80 RPM, and are encouraged to continue exercising until exhaustion, or inability to maintain the pedal frequency of 70 RPM. Expired gases are collected and analyzed breath by breath for O_2_, CO_2_, and volume. The average values of the last 30 seconds of exercise are used as measures for peak oxygen uptake (peakVO_2_, in l/min), peak power output (peakW, in watt), and peak HR. Ventilatory threshold is determined by the oxygen equivalent method [30], using the average value obtained by two independent observers. HR and respiratory exchange ratio (RER) are used as objective criteria for peak exercise.

#### Muscle strength

Upper extremity muscle strength is assessed by using a JAMAR grip strength dynamometer. Each patient is asked to grip first right-handed then left-handed three consecutive times. The maximum score in terms of kilograms is recorded for each side. Handgrip dynamometry can be used to characterize general upper limb muscle strength dynamometer [31-33]. Handgrip strength can increase after general resistance training of the upper extremities, consisting of exercises that did not specifically involve hand grip strength [34].

Lower extremity muscle strength is assessed by the 30 seconds chair stand test [35]. The patient is asked to stand upright from a chair with their arms folded across the chest, then to sit down again and then repeat the action at his or her fastest pace over a 30 seconds period. The final test score is the number of times that the subject rises to a full stand [35,36]. The 30 seconds chair stand test is a valid and reliable measure of proximal lower limb strength in older adults [37].

#### Fatigue

Two self-report questionnaires are used to assess fatigue: the Multidimensional Fatigue Inventory (MFI) [38,39] and the Fatigue Quality List (FQL) [40].

The MFI is a questionnaire consisting of 20 statements for which the person has to indicate on a 0-5 scale to what extent the particular statement applies to him or her. This self-report instrument consists of five subscales based on different dimensions: general fatigue, physical fatigue, reduced physical activity, reduced motivation and mental fatigue. The MFI subscales have exhibited adequate reliability for purposes of group comparisons and has good known group validity [38].

The patients' perception and appraisal of experienced fatigue is assessed with the FQL. The FQL consists of 25 adjectives describing the fatigue experience, organized into four subscales: frustrating, exhausting, pleasant, and frightening.

### Secondary outcome measures

The REACT study assesses the following secondary outcome measures: health-related quality of life, body composition, bone mineral density, neuropathy, objective and self-reported daily physical activity level, mood and sleep disturbances, functioning in daily life, return to work, cost from a social perspective, adverse events, compliance and satisfaction with the intervention. In addition, clinical data, disease status and treatment, sociodemographic characteristics, moderating variables of the exercise programme and adverse events will be recorded. A complete overview of primary and secondary outcome measures is provided in Table 1. A small selection of these secondary measures is described in detail below. A detailed description of the secondary outcome measures, common to all four trials, are described in an overall design paper [25].

**Table 1:** Overview Primary and Secondary outcome measures

Outcome measures	Instrument
**A. Primary outcome measures**	

Cardiorespiratory fitness	Maximum exercise test (peakVO_2 _)

Muscular strength	30 s sit-to-stand test, maximal handgrip strength

Fatigue	Multidimensional Fatigue Inventory (MFI) [38] and the Fatigue Quality List (FQL) [40] questionnaires

**B. Secondary outcome measures**	

Sociodemographic data	Age, education, marital status, living situation, comorbidities and life style variables (e.g. smoking)

Clinical data	

Medical history	Date of diagnosis, subtype of disease, stage of disease, history of therapy

Disease status and treatment	Response to treatment, progression or relapse of disease and data on any additional treatment will be recorded from medical records

Adverse events	Medical records, reports of the sports physician and physical therapist

Physical tests	

Physical examination	Height, weight, waist and hip circumferences, four skinfolds (biceps, triceps, suprailiacal and subscapular)

Body composition and bone mineral density	Dual Energy X-ray (DXA) scan

Questionnaires	

Health-related quality of life	EORTC Quality of Life Questionnaire C30 (EORTC QLQ-C30) [43], EuroQol (EQ5D) [44], EORTC Chemotherapy-induced peripheral neuropathy module (QLQ-CIPN20) [45]

Physical activity	Physical Activity Scale for the Elderly (PASE) [46], Recordings of the Actitrainer accelerometer (Actigraph, Fort Walton Beach Florida, USA)

Mood disturbance	Hospital Anxiety and Depression Scale (HADS) [47,48]

Functioning in daily life	Impact on Participation and Autonomy (IPA) [49]

Quality of Sleep	Pittsburgh Sleep Quality Index (PSQI) [50]

Return to work	Return to work questionnaire

Moderating variables	Questionnaire about pre-illness lifestyle, current attitudes toward and beliefs about exercise in general

Satisfaction with the intervention	Satisfaction questionnaire

Cost questionnaires	Cost dairies

Compliance with the exercise program	Self-report and objective measures (e.g. attendance, exercise logs, target intensity)

#### Sociodemographic and clinical data

Sociodemographic data such as age, level of education, marital status, living situation, medication use (including alternative medications or therapies) and lifestyle variables (e.g. smoking) are obtained by questionnaire.

Clinical information, including date of diagnosis, stage and subtype of disease, and treatment history is obtained from medical records. During the follow-up period, data on disease status (response to treatment, progression or relapse) and data on any additional treatment are collected.

#### Moderating variables

At baseline, a series of questions is used to assess a number of potential moderating variables, including pre-illness lifestyle (frequency, nature and intensity of daily physical activity and exercise behaviour, or avoidance thereof), current attitudes towards and beliefs about exercise and daily physical activity in general, and about exercising after chemotherapy. These questions are adapted from measures developed by Courneya and colleagues [41,42] for use in evaluating exercise in cancer survivors, and are based on established health behaviour theories, in particular the Theory of Planned Behaviour [42].

#### Costs from a societal perspective

Besides the costs of the exercise programmes, data on health care costs, patient and family costs, and costs of production losses are collected using cost diaries administered on a 3-monthly basis during the entire follow-up period. Health care costs include the costs of oncological care, general practice care and physiotherapy, additional visits to other health care providers, prescription of medication, professional home care and hospitalization. Patient and family costs include out-of-the-pocket expenses such as travel expenses, over-the-counter medication, and costs for paid and unpaid help. Costs related to production losses include work absenteeism for patients with paid jobs, and days of inactivity for patients without a paid job.

### Power calculations

Power calculation is based on the effects on physical fitness and fatigue found in the study by De Backer et al. [13] examining the long-term effects of a high intensity resistance and endurance exercise programme after cancer treatment compared with natural recovery.

With a sample size of 80 we are able to detect a difference in fatigue of 9 points (EORTC QLQ-C30), with a standard deviation (SD) of 20, a power of 0.80 and two sided alpha of 0.05. Additional power calculations (a power of 0.80 and alpha of 0.05) for muscle strength (vertical row) and cardiorespiratory fitness (peakVO_2_) showed that this sample size enables us to detect a difference in vertical row of 0.09 1-RM/kg (SD 0.20) and a difference in peakVO_2 _of 3 ml/min/kg (SD 7). Both supplementary calculations are based on differences in results reported by the same research group [13].

To compensate for dropouts and taking into account the multi-level design we aim to enrol 40% more patients, therefore in total 120 subjects per group. Since we expect smaller differences between the high intensity exercise group and the low-to-moderate intensity exercise group, we have decided to enlarge these two groups to 140 subjects per group. The waiting list control group will consist of 120 patients.

### Statistical analysis

Baseline characteristics of the two intervention groups and control group with regard to the most important prognostic indicators and main outcome measures will be compared to assess the adequacy of the randomization. If necessary, adjustments will be made for baseline characteristics. In a similar way, we will assess differences between responders and non-responders with regard to the most important prognostic indicators in order to describe the generalizability of the results.

Data are analyzed according to the intention-to-treat principle. In addition, per protocol analysis will be performed, in which only patients will be included who attained 75% of all exercise sessions.

Scores on the self-report measures of fatigue, mood state and health-related quality of life will be calculated according to published scoring algorithms. Multilevel longitudinal regression analysis will be conducted to assess changes in each outcome measure. The follow-up measurements will be defined as dependent variable and the following levels are used, 1) time of follow-up measurement (values corresponding with performance at t1 and t2); 2) training centre 3) individual. Regression coefficients will indicate differences between interventions and control group. Regression models will be adjusted for gender, baseline values, and compliance. Missing values will be avoided as much as possible by asking participants to comply with the post-treatment and follow-up measurement even after they drop out from the exercise programme. In the event of missing values, the mixed linear regression modelling will account for them.

### Cost-effectiveness analysis

The economic evaluation includes cost-effectiveness and cost-utility analysis from a societal perspective, and will be performed according to the intention-to-treat principle. Detailed descriptions of the economic evaluation are described in the overall design paper [25].

## Discussion

This project aims to contribute to evidence-based practice in cancer rehabilitation programmes. We evaluate the effectiveness of exercise in cancer patients with respect to improving physical fitness and reducing fatigue. Preliminary results in the literature are promising. Yet, the suggested positive physical and psychosocial outcomes of exercise programmes among cancer patients need to be confirmed in large, well-designed trials.

The REACT study evaluates the effectiveness and cost-effectiveness of a high intensity exercise programme compared to a low-to-moderate exercise programme, and a waiting list control group. In this way, we will obtain more insight in outcomes of different training intensities. Furthermore, if exercise appears effective, it becomes vital to evaluate cost-effectiveness and cost savings for health care utilization since exercise-based rehabilitation programmes do not yet form part of standard cancer care for cancer patients and survivors.

The following suggestions made by the systematic reviews have been incorporated to strengthen our methodology; applying a larger sample and long-term follow-up measurements, incorporating randomization of patients to appropriate comparison groups, including concealed allocation and blinded outcome assessment, and using intention-to-treat and per protocol analyses. Most studies so far have focused on breast cancer patients [16-18]. The inclusion criteria of the present study include as many as four cancer diagnoses (primary breast, colon or ovarian cancer, or lymphomas) allowing to explore whether patients with different cancer types respond differently to exercise. Furthermore, detailed evaluation of current attitudes towards and beliefs about exercise of both responders and non-responders are obtained. This provides insight concerning the generalizability of the results from this RCT.

Limitations of the study should be noted as well. Instead of a 'true' non-exercising control group, the present study includes a waiting list control group. Due to the growing availability of cancer rehabilitation groups in daily clinical practices in the study region, patients' expectations may not be met, and higher drop-out rates could occur with a non-exercising control group. However, the use of a waiting list control group does not allow long-term follow-up measurements, because after 12 weeks these patients start the exercise programme they have been allocated to.

The prescribed design of resistance and endurance interval exercises in the present study is originally based on the intervention assessed by De Backer et al. (2007) [9]. To improve the earlier exercise programme and to be more closely aligned to the ACSM guidelines [23], we added an additional endurance interval session with a constant workload. Hence, the resulting combination of exercises is in our opinion state-of-the-art, included in a firm study design.

## Competing interests

The authors declare that they have no competing interests.

## Authors' contributions

All authors were involved in the design of the study. LB, GS, WM, JB and MC are the principal investigators of this study. CK is conducting this research in fulfillment of a PhD, and will be responsible for data collection, analysis and interpretation. All authors have read and approved the final version of the manuscript.

## Pre-publication history

The pre-publication history for this paper can be accessed here:

http://www.biomedcentral.com/1471-2407/10/658/prepub

## References

[B1] iKCnet - Kankerregistratie http://ikcnet.nl

[B2] Pihkala J Happonen JM Virtanen K Sovijarvi A Siimes MA Pesonen E Cardiopulmonary evaluation of exercise tolerance after chest irradiation and anticancer chemotherapy in children and adolescents Pediatrics 1995 95 722 726 7724311

[B3] Lucia A Earnest C Perez M Cancer-related fatigue: can exercise physiology assist oncologists? Lancet Oncol 2003 4 616 625 10.1016/S1470-2045(03)01221-X 14554239

[B4] Jones JM Cheng T Jackman M Rodin G Walton T Catton P Self-efficacy, perceived preparedness, and psychological distress in women completing primary treatment for breast cancer J Psychosoc Oncol 2010 28 269 290 10.1080/07347331003678352 20432117

[B5] Curt GA Breitbart W Cella D Groopman JE Horning SJ Itri LM Impact of cancer-related fatigue on the lives of patients: new findings from the Fatigue Coalition Oncologist 2000 5 353 360 10.1634/theoncologist.5-5-353 11040270

[B6] de Jong N Courtens AM bu-Saad HH Schouten HC Fatigue in patients with breast cancer receiving adjuvant chemotherapy: a review of the literature Cancer Nurs 2002 25 283 297 10.1097/00002820-200208000-00004 12181497

[B7] Broeckel JA Jacobsen PB Horton J Balducci L Lyman GH Characteristics and correlates of fatigue after adjuvant chemotherapy for breast cancer J Clin Oncol 1998 16 1689 1696 9586880 10.1200/JCO.1998.16.5.1689

[B8] Dimeo FC Effects of exercise on cancer-related fatigue Cancer 2001 92 1689 1693 10.1002/1097-0142(20010915)92:6+<1689::AID-CNCR1498>3.0.CO;2-H 11598888

[B9] van Weert E Hoekstra-Weebers JE May AM Korstjens I Ros WJ van der Schans CP The development of an evidence-based physical self-management rehabilitation programme for cancer survivors Patient Educ Couns 2008 71 169 190 10.1016/j.pec.2007.11.027 18255249

[B10] De Backer I van Breda E Vreugdenhil A Nijziel MR Kester AD Schep G High-intensity strength training improves quality of life in cancer survivors Acta Oncol 2007 46 1143 1151 10.1080/02841860701418838 17851864

[B11] May AM van Weert E Korstjens I Hoekstra-Weebers JE van der Schans CP Zonderland ML Improved physical fitness of cancer survivors: a randomised controlled trial comparing physical training with physical and cognitive-behavioural training Acta Oncol 2008 47 825 834 10.1080/02841860701666063 17917819

[B12] May AM Korstjens I van WE van den BB Hoekstra-Weebers JE van der Schans CP Long-term effects on cancer survivors' quality of life of physical training versus physical training combined with cognitive-behavioral therapy: results from a randomized trial Support Care Cancer 2009 17 653 663 10.1007/s00520-008-0519-9 18953578

[B13] De Backer I Vreugdenhil G Nijziel MR Kester AD van Breda E Schep G Long-term follow-up after cancer rehabilitation using high-intensity resistance training: persistent improvement of physical performance and quality of life Br J Cancer 2008 99 30 36 10.1038/sj.bjc.6604433 18577993 PMC2453017

[B14] Knols R Aaronson NK Uebelhart D Fransen J Aufdemkampe G Physical exercise in cancer patients during and after medical treatment: a systematic review of randomized and controlled clinical trials J Clin Oncol 2005 23 3830 3842 10.1200/JCO.2005.02.148 15923576

[B15] Galvao DA Newton RU Review of exercise intervention studies in cancer patients J Clin Oncol 2005 23 899 909 10.1200/JCO.2005.06.085 15681536

[B16] Cramp F Daniel J Exercise for the management of cancer-related fatigue in adults Cochrane Database Syst Rev 2008 CD006145 18425939 10.1002/14651858.CD006145.pub2

[B17] Courneya KS Exercise in cancer survivors: an overview of research Med Sci Sports Exerc 2003 35 1846 1852 10.1249/01.MSS.0000093622.41587.B6 14600549

[B18] Stevinson C Lawlor DA Fox KR Exercise interventions for cancer patients: systematic review of controlled trials Cancer Causes Control 2004 15 1035 1056 10.1007/s10552-004-1325-4 15801488

[B19] Segal RJ Reid RD Courneya KS Malone SC Parliament MB Scott CG Resistance exercise in men receiving androgen deprivation therapy for prostate cancer J Clin Oncol 2003 21 1653 1659 10.1200/JCO.2003.09.534 12721238

[B20] De Backer I Schep G Backx FJ Vreugdenhil G Kuipers H Resistance Training in Cancer Survivors: A Systematic Review Int J Sports Med 2009 19585401 10.1055/s-0029-1225330

[B21] Courneya KS Segal RJ Mackey JR Gelmon K Reid RD Friedenreich CM Effects of aerobic and resistance exercise in breast cancer patients receiving adjuvant chemotherapy: a multicenter randomized controlled trial J Clin Oncol 2007 25 4396 4404 10.1200/JCO.2006.08.2024 17785708

[B22] Schmitz KH Courneya KS Matthews C mark-Wahnefried W Galvao DA Pinto BM American College of Sports Medicine roundtable on exercise guidelines for cancer survivors Med Sci Sports Exerc 2010 42 1409 1426 10.1249/MSS.0b013e3181e0c112 20559064

[B23] Thompson WR Gordon NF Pescatello LS ACSM's Guidelines for exercise training and prescribtion 2009 8

[B24] Wisloff U Stoylen A Loennechen JP Bruvold M Rognmo O Haram PM Superior cardiovascular effect of aerobic interval training versus moderate continuous training in heart failure patients: a randomized study Circulation 2007 115 3086 3094 10.1161/CIRCULATIONAHA.106.675041 17548726

[B25] Chinapaw MJM Buffart LM van Mechelen W Schep G Aaronson NK van Harten WH Stuiver MM Kersten MJ Nollet F Kaspers GJL van Dulmen-den Broeder E Huisman J Takken T van Tulder M Brug J Alpe d'HuZes Cancer Rehabilitation (A-CaRe) research: Four randomized controlled exercise trials and economic evaluations in cancer patients and survivors Submitted 10.1007/s12529-011-9158-5 PMC3358561 21556821

[B26] Levinger I Goodman C Hare DL Jerums G Toia D Selig S The reliability of the 1RM strength test for untrained middle-aged individuals J Sci Med Sport 2009 12 310 316 10.1016/j.jsams.2007.10.007 18078784

[B27] De Backer I Schep G Hoogeveen A Vreugdenhil G Kester AD van Breda E Exercise testing and training in a cancer rehabilitation program: the advantage of the steep ramp test Arch Phys Med Rehabil 2007 88 610 616 10.1016/j.apmr.2007.02.013 17466730

[B28] Karvonen J Vuorimaa T Heart rate and exercise intensity during sports activities. Practical application Sports Med 1988 5 303 311 10.2165/00007256-198805050-00002 3387734

[B29] Clinical exercise testing with reference to lung diseases: indications, standardization and interpretation strategies. ERS Task Force on Standardization of Clinical Exercise Testing. European Respiratory Society Eur Respir J 1997 10 2662 2689 10.1183/09031936.97.10112662 9426113

[B30] Wasserman K Critical capillary PO2 and the role of lactate production in oxyhemoglobin dissociation during exercise Adv Exp Med Biol 1999 471 321 333 10659163 10.1007/978-1-4615-4717-4_39

[B31] Bohannon RW Hand-grip dynamometry provides a valid indication of upper extremity strength impairment in home care patients J Hand Ther 1998 11 258 260 9862263 10.1016/s0894-1130(98)80021-5

[B32] Bohannon RW Is it legitimate to characterize muscle strength using a limited number of measures? J Strength Cond Res 2008 22 166 173 10.1519/JSC.0b013e31815f993d 18296971

[B33] Bohannon RW Dynamometer measurements of grip and knee extension strength: are they indicative of overall limb and trunk muscle strength? Percept Mot Skills 2009 108 339 342 10.2466/pms.108.2.339-342 19544938

[B34] Magnussen Thomas E Sahlberg M Svantesson U The effect of resistance training on handgrip strength in young adults Isokinetics and Exercise Science 2009 16 125 131

[B35] Jones CJ Rikli RE Beam WC A 30-s chair-stand test as a measure of lower body strength in community-residing older adults Res Q Exerc Sport 1999 70 113 119 10380242 10.1080/02701367.1999.10608028

[B36] Csuka M McCarty DJ Simple method for measurement of lower extremity muscle strength Am J Med 1985 78 77 81 10.1016/0002-9343(85)90465-6 3966492

[B37] Rikli RE Jones CJ Functional fitness normative scores for community-residing older adults, ages 60-94 J Aging Phys Activity 1999 7 162 181

[B38] Smets EM Garssen B Bonke B de Haes JC The Multidimensional Fatigue Inventory (MFI) psychometric qualities of an instrument to assess fatigue J Psychosom Res 1995 39 315 325 10.1016/0022-3999(94)00125-O 7636775

[B39] Echteld MA Passchier J Teunissen S Claessen S de Wit R van der Rijt CC Multidimensional fatigue and its correlates in hospitalised advanced cancer patients Eur J Cancer 2007 43 1030 1036 10.1016/j.ejca.2007.01.024 17336052

[B40] Gielissen MF Knoop H Servaes P Kalkman JS Huibers MJ Verhagen S Differences in the experience of fatigue in patients and healthy controls: patients' descriptions Health Qual Life Outcomes 2007 5 36 10.1186/1477-7525-5-36 17605783 PMC1934901

[B41] Courneya KS Understanding readiness for regular physical activity in older individuals: an application of the theory of planned behavior Health Psychol 1995 14 80 87 10.1037/0278-6133.14.1.80 7737078

[B42] Courneya KS Friedenreich CM Utility of the theory of planned behavior for understanding exercise during breast cancer treatment Psychooncology 1999 8 112 122 10.1002/(SICI)1099-1611(199903/04)8:2<112::AID-PON341>3.0.CO;2-L 10335555

[B43] Aaronson NK Ahmedzai S Bergman B Bullinger M Cull A Duez NJ The European Organization for Research and Treatment of Cancer QLQ-C30: a quality-of-life instrument for use in international clinical trials in oncology J Natl Cancer Inst 1993 85 365 376 10.1093/jnci/85.5.365 8433390

[B44] Kopec JA Willison KD A comparative review of four preference-weighted measures of health-related quality of life J Clin Epidemiol 2003 56 317 325 10.1016/S0895-4356(02)00609-1 12767408

[B45] Postma TJ Aaronson NK Heimans JJ Muller MJ Hildebrand JG Delattre JY The development of an EORTC quality of life questionnaire to assess chemotherapy-induced peripheral neuropathy: the QLQ-CIPN20 Eur J Cancer 2005 41 1135 1139 10.1016/j.ejca.2005.02.012 15911236

[B46] Washburn RA Smith KW Jette AM Janney CA The Physical Activity Scale for the Elderly (PASE): development and evaluation J Clin Epidemiol 1993 46 153 162 10.1016/0895-4356(93)90053-4 8437031

[B47] Spinhoven P Ormel J Sloekers PP Kempen GI Speckens AE van Hemert AM A validation study of the Hospital Anxiety and Depression Scale (HADS) in different groups of Dutch subjects Psychol Med 1997 27 363 370 10.1017/S0033291796004382 9089829

[B48] Zigmond AS Snaith RP The hospital anxiety and depression scale Acta Psychiatr Scand 1983 67 361 370 10.1111/j.1600-0447.1983.tb09716.x 6880820

[B49] Cardol M Beelen A van den Bos GA De Jong BA de G de Haan RJ Responsiveness of the Impact on Participation and Autonomy questionnaire Arch Phys Med Rehabil 2002 83 1524 1529 10.1053/apmr.2002.35099 12422319

[B50] Buysse DJ Reynolds CF Monk TH Berman SR Kupfer DJ The Pittsburgh Sleep Quality Index: a new instrument for psychiatric practice and research Psychiatry Res 1989 28 193 213 10.1016/0165-1781(89)90047-4 2748771

